# Reconsidering palliative radiotherapy in addition to PD-1 blockade for non-small cell lung cancer: results from the FORCE phase II trial (AIO/YMO-TRK-0415)

**DOI:** 10.1007/s10585-025-10358-x

**Published:** 2025-07-24

**Authors:** Farastuk Bozorgmehr, Inn Chung, Jürgen R. Fischer, Marc Bischof, Akin Atmaca, Sophie Wetzel, Martin Faehling, Dirk Bottke, Martin Wermke, Esther G. C. Troost, Cornelia Kropf-Sanchen, Thomas Wiegel, Bernd Schmidt, Andrej Stupavsky, Walburga Engel-Riedel, Michaela Hammer-Hellmig, Niels Reinmuth, Farkhad Manapov, Christian Grohe, Robert Krempien, Christian Schumann, Florian Sterzing, Martin Reck, Florian Würschmidt, Jochen Fleckenstein, Alev Petroff, Sven Henschke, Rouven Behnisch, Jelena Cvetkovic, Lena Brückner, Constantin Schwab, Albrecht Stenzinger, Thorsten Götze, Christina Kopp, Helge Schröder, Jürgen Debus, Petros Christopoulos, Michael Thomas, Stefan Rieken

**Affiliations:** 1 Department of Thoracic Oncology, Thoraxklinik, Heidelberg University Hospital and National Center for Tumor Diseases (NCT), NCT Heidelberg, a partnership between DKFZ and Heidelberg University Hospital, Heidelberg, Germany; 2 Translational Lung Research Center Heidelberg TLRC-H, Member of the German Center for Lung Research (DZL), Heidelberg, Germany; 3 Department of Thoracic Oncology, Lungenklinik Loewenstein, Loewenstein, Germany; 4 Department of Radiation Oncology, Klinikum Am Gesundbrunnen, SLK-Kliniken Heilbronn GmbH, Heilbronn, Germany; 5 Department of Hematology and Oncology, Krankenhaus Nordwest, UCT-University Cancer Center, Frankfurt, Germany; 6 Department of Radiation Oncology, Krankenhaus Nordwest, Frankfurt, Germany; 7 Department of Cardiology and Pneumology, Klinikum Esslingen, Esslingen, Germany; 8 Department of Radiation Oncology, MVZ Klinikum Esslingen, Esslingen, Germany; 9 Medizinische Fakultät Carl-Gustav-Carus der Technischen Universität, Medizinische Klinik Und Poliklinik I, University Hospital Carl-Gustav-Carus, National Center for Tumor Diseases (NCT)-Partner Site Dresden, Medizinische Fakultät der Technischen Universität, Universitätskrebscentrum, Early Clinical Trial Unit, German Cancer Consortium (DKTK), Partner Site Dresden, Dresden, Germany; 10 Department of Radiotherapy and Radiation Oncology, Faculty of Medicine and University Hospital Carl Gustav Carus, Technische Universität Dresden, OncoRay - National Center for Radiation Research in Oncology, Dresden, Germany; 11 Department of Pneumology, University Hospital Ulm, Ulm, Germany; 12 Department of Radiotherapy and Radiation Oncology, University Hospital Ulm, Ulm, Germany; 13 Department of Internal Medicine, Pneumology and Sleep Medicine, DRK Kliniken Berlin Mitte, Berlin, Germany; 14 Department of Radiation Oncology, DRK Kliniken Berlin Westend, Berlin, Germany; 15 Department of Pneumology, City of Cologne Municipal Hospitals, Lung Hospital Cologne Merheim, Cologne, Germany; 16 Department of Radiation Oncology, City of Cologne Municipal Hospitals, Cologne, Germany; 17 Department of Thoracic Oncology, Asklepios Fachkliniken München-Gauting, Member of the German Center for Lung Research (DZL), Gauting, Germany; 18 Department of Radiation Oncology, University Hospital Munich (LMU), Munich, Germany; 19 Department of Pneumology, Evangelische Lungenklinik Berlin, Berlin, Germany; 20 Department of Radiation Oncology, Helios Klinikum Berlin-Buch, Berlin, Germany; 21 Clinic for Pneumology, Thoracic Oncology, Sleep, and Respiratory Critical Care, Allgäu Clinics, Kempten and Immenstadt, Immenstadt, Germany; 22 Department of Radiation Oncology, Klinikum Kempten, Kempten, Germany; 23 Department of Thoracic Oncology, LungenClinic Grosshansdorf, Airway Research Centre North (ARCN), Member of the German Center for Lung Research (DZL), Grosshansdorf, Germany; 24 Radiologische Allianz, Hamburg, Germany; 25 Department of Radiotherapy and Radiation Oncology, Saarland University Medical Center, Homburg, Germany; 26 Internal Medicine V, Saarland University Medical Center, Homburg, Germany; 27 Institute of Medical Biometry, Heidelberg University, Heidelberg, Germany; 28 Institute of Pathology, Heidelberg University Hospital, Heidelberg, Germany; 29 Institut Für Klinische Krebsforschung IKF GmbH Am Krankenhaus Nordwest, Frankfurt, Germany; 30 AIO-Studien gGmbH, Berlin, Germany; 31 Department of Radiation Oncology, Heidelberg University Hospital, Heidelberg, Germany; 32 Department of Radiotherapy and Radiation Oncology, University Medical Center Goettingen, Goettingen, Germany

**Keywords:** Non-small cell lung cancer, Immunotherapy, Radioimmunotherapy, Nivolumab, Palliative radiotherapy

## Abstract

Introduction of immune checkpoint inhibitors (ICI) has improved overall survival (OS) for advanced non-small cell lung cancer (NSCLC). However, responses differ greatly amongst patients. Additional radiotherapy (RT) may promote tumor-specific immunity and synergize with ICI to improve tumor control. The multicenter phase II FORCE trial evaluated safety and efficacy of nivolumab with additional palliative radiotherapy (5 × 4 Gy) as clinically indicated in pre-treated metastatic non-squamous NSCLC (group A, *n* = 41), including pretreated patients without an indication for radiotherapy in a parallel cohort as real-world controls (group B, *n* = 60). With an objective response rate (ORR) of 8.3% in group A (*n* = 41), the primary endpoint was not met (*p* = 0.991). ORR in group B (*n* = 60) was 23.8%. Patient characteristics indicated a significantly poorer baseline clinical condition for group A compared to B, including worse Eastern Cooperative Oncology Group (ECOG) performance status (PS, *p* = 0.020) and more metastatic sites (*p* = 0.009). Consequently, group A had shorter progression-free survival (median PFS, 1.9 versus 3.7 months, hazard ratio [HR] 1.69 [95% CI (1.10, 2.58)]) and OS (median 6.0 versus 12.6 months, HR 1.75 [95% CI (1.07, 2.84)]). In multivariable analyses for the intention-to-treat (ITT) population, ORR, PFS and OS were inversely associated with the patients’ ECOG PS (ORR odds ratio [OR] 0.126, *p* = 0.004) and correlated positively with tumor PD-L1 expression (ORR OR 12.8, *p* = 0.022), but not with radiotherapy administration (*p* = 0.169–0.854). Adverse events were distributed equally in both groups. Addition of palliative radiotherapy to nivolumab was safe and feasible, but not associated with improved efficacy. Patients with an indication for palliative radiotherapy have an inherently worse prognosis which cannot be overcome by radiation-induced immunostimulation. Clinical features and PD-L1 expression influence clinical outcomes more than radiotherapy administration and should be considered when evaluating the effectiveness of immuno-/radiotherapy combinations.

*ClinicalTrials.gov identifier*: NCT03044626

**Supplementary Information:**

The online version contains supplementary material available at 10.1007/s10585-025-10358-x.

## Background

Despite major treatment advances in recent years, non-small cell lung cancer (NSCLC) is still a leading cause of cancer-related death, with most patients diagnosed at an advanced, incurable stage [1]. In particular, PD-1/PD-L1 immune checkpoint inhibitors (ICI) have improved OS and meanwhile represent a new standard of care [2–5], as they result in longer remissions at a generally better tolerability compared to chemotherapy alone. However, a significant percentage of patients do not respond and 5–10% even experience tumor hyperprogression [4, 6–9], which creates a pressing need for novel strategies to augment ICI efficacy.

Radiotherapy (RT), commonly applied to relieve local symptoms, may also induce tumor-specific immune effects, both inside and outside the irradiated field, a process known as the abscopal effect [10–17]. Given these immune-stimulating effects of irradiation, a combination with ICI may synergistically boost immune-mediated tumor cell death and thus potentially improve response rates. While radiotherapy alone is rarely sufficient to elicit an abscopal effect in clinical practice [13, 18, 19], photon radiotherapy applied prior to immunotherapy has been shown to significantly improve survival irrespective of PD-L1 expression, and a meta-analysis found improved OS and PFS for advanced NSCLC patients treated with ICI-radiotherapy combination compared to ICI or radiotherapy alone, providing clinical evidence for a synergistic immune stimulatory effect [20, 21]. In addition, several case reports describing responses in metastatic lesions distant from the irradiated field in NSCLC patients undergoing ICI therapy highlight the relevance of the abscopal effect in clinical practice [22–24]. How the synergistic effect of the combination between immune- and radiotherapy can be best employed and which parameters play a role in this regard are a field of active research [25].

The prospective FORCE study was designed to investigate the potential synergy between ionizing radiation and ICI for enhancement of anti-tumor immunity in NSCLC including pretreated patients without an indication for radiotherapy in a parallel cohort as real-world controls. To our knowledge, this is the first prospective clinical interventional trial to analyze the potential impact of palliative radiotherapy on tumor control for NSCLC patients with direct applicability in the real-world setting.

## Methods

### Patients

Patients with metastatic non-squamous NSCLC after failure of platinum-based chemotherapy were included from February 2017 to December 2019. Key inclusion criteria were age ≥ 18 years, availability of paraffin-embedded tissue blocks for evaluation of PD-L1 expression, and adequate clinical performance (ECOG PS 0–1). A detailed description of inclusion criteria can be found in the published protocol [26]. Enrolled patients were non-randomly allocated to group A or group B based on their indication for radiotherapy. Specifically, patients with a clinical indication for palliative radiotherapy to metastatic sites other than the lung and brain (e.g. for bone: pain relief, prevention of pathological fracture or stabilization, prevention of spinal cord compression; lymph nodes: local compression; skin: ulcerating lesions) as well as at least one measurable, not previously irradiated lesion to facilitate evaluation of efficacy, were allocated to group A and received nivolumab combined with radiotherapy. Patients without an indication for radiotherapy were allocated to group B and received nivolumab monotherapy serving as a real-world control cohort.

### Trial design

The FORCE trial (ClinicalTrials.gov identifier: NCT03044626) was a prospective, multicenter, interventional two-group, non-randomized, open-label phase II study investigating the feasibility, efficacy, and safety of nivolumab treatment combined with radiotherapy in metastatic non-squamous NSCLC [26]. In group A, radiotherapy was initiated 72 h after the initial nivolumab administration (240 mg) and applied in five fractions (20 Gy in total, 5 × 4 Gy).

Details of radiotherapy planning have been published before [26]. In short, radiotherapy planning was CT-based. To ensure robust and reproducible patient positioning during both planning and treatment, positioning aids (e.g., masks, cushions, vacuum beds) were allowed. All non-cerebral and non-pulmonary tumor lesions could be included into the trial and were irradiated if clinically indicated. If the planned target volume (PTV) did not directly overlap with the contoured lungs, patients with mediastinal lymph nodes or thoracic bone and soft tissue lesions (such as the thoracic spine or chest wall) could be included.

Gross tumor volumes (GTV) were contoured on planning CTs, incorporating co-registered imaging (e.g., MRI, PET) if available. Clinical (CTV) and planning target volumes (PTV) were derived using institutional margins to ensure coverage while sparing organs at risk (OAR).

Treatment was delivered on workdays in 5 fractions of 4 Gy (total dose: 20 Gy). Dose specification followed the International Commission of Radiation Units and Measurements (ICRU) reports 50, 62, and 83.

Nivolumab was administered at 240 mg fixed dose every two weeks in both groups until disease progression (according to response evaluation criteria in solid tumors (RECIST) v1.1), loss of clinical benefit, or occurrence of unacceptable toxicity. Clinical assessments were carried out as previously described [26]. Tumor staging was performed at baseline and every 6 weeks for the first year starting at week 9 after therapy initiation. In the second year, the frequency of restaging was extended to every 12 weeks until disease progression.

Assessment of PD-L1 expression with the tumor proportion score (TPS) is described in the Supplementary Methods.

### Endpoints

The primary efficacy endpoint was the ORR defined as best overall response (BOR) from baseline being either complete or partial response (CR or PR) according to RECIST v1.1. Secondary endpoints were PFS and OS, as well as toxicity and quality of life (QoL) assessed via FACT-L (see also Supplementary Methods).

### Statistical design and analysis

Statistical analysis followed the International Conference on Harmonization Guidelines “Structure and Content of Clinical Study Reports” and “Statistical Principles for Clinical Trials”. Expected response rates were based on the results of Checkmate-057 trial with an ORR of 19% in nivolumab-treated patients, [5] whose significant improvement at 35% upon combination with radiotherapy would be detectable with a power of 80% using a binomial test with one-sided significance of α = 0.05.

For the primary endpoint ORR, one-sided Agresti-Coull 95%-confidence intervals were provided in both groups. Median survival times and 1-year rates were calculated for the secondary endpoints OS and PFS which were compared with logrank tests. PFS and OS were estimated by the Kaplan–Meier method from the time point of first nivolumab administration. Cox and logistic regression were used to examine the effect of covariates, e.g. PD-L1 TPS, on survival endpoints and ORR. As PD-L1 TPS was missing for 7 patients, the sample size for these analyses was reduced to 89 cases.

Patient-reported outcomes (PRO) and QoL were assessed via a linear mixed model with the dependent variable QoL at follow-up, taking the QoL at baseline and treatment group as fixed factors and time since start of therapy per patient as a repeated factor into account. The dependency between QoL measurements at follow-up was modelled using a continuous auto-regressive order 1 covariance matrix.

Safety analyses included all patients who received at least one dose of study treatment with tabulation of relative and absolute frequencies for (serious) adverse events ([S]AEs) according to CTCAE version 4.03.

Due to the change of standard therapy with movement of ICI from the second to the first line of treatment during this study [3, 27], the trial did not achieve the anticipated number of 50 patients with evaluable primary outcome in group A. Statistical analysis was performed as specified in the study protocol and statistical analysis plan (SAP). Additional exploratory analyses according to PD-L1 expression, clinical covariates and the irradiated sites were performed using the statistical methods described above to adjust for potential confounders.

## Results

### Patient and treatment characteristics

**Fig. 1:**
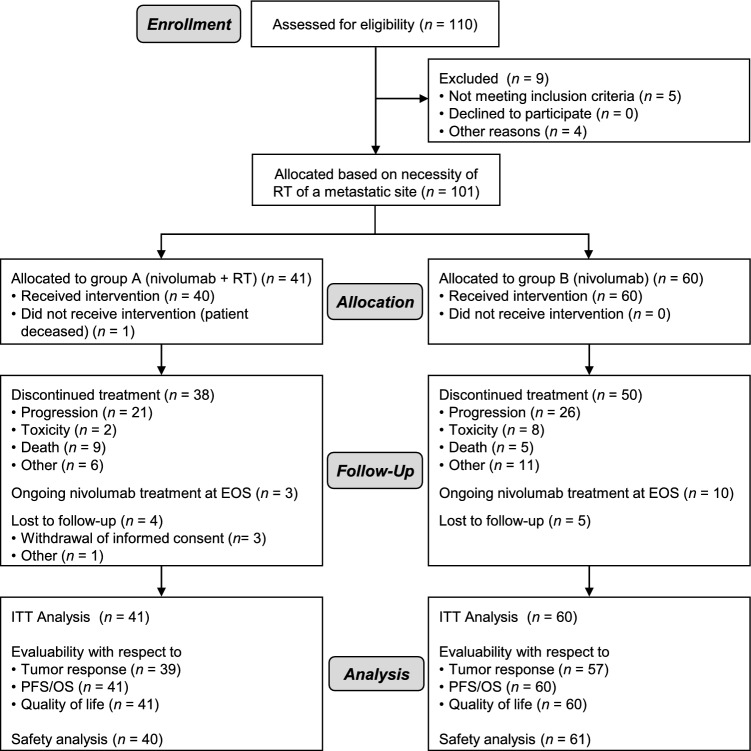
CONSORT diagram of patient distribution in the FORCE trial. One patient allocated to group A received nivolumab only and was assigned to group B for safety analysis. EOS, end of study; ITT, intention-to-treat; OS, overall survival; PFS, progression-free survival; RT, radiotherapy

From February 2017 to December 2019, a total of 101 patients from 16 participating centers across Germany were enrolled and non-randomly allocated to group A (nivolumab plus radiotherapy, *n* = 41) or group B (nivolumab only, *n* = 60) based on their clinical indication for palliative radiotherapy allowing for a descriptive statistical analysis of all study endpoints. All patients in the study population had reached the end of protocol treatment (EOT) at the time of the final analysis (data cut-off May 7th 2021). The reason for EOT was progressive disease in 21 patients (51.2%) treated in group A and in 26 patients (43.3%) in group B (Fig. 1). Three patients from group A and ten patients from group B continued to receive nivolumab after the end of study (EOS). The primary analysis for all efficacy outcomes was conducted for the intention-to-treat (ITT) population (Fig. 1). For evaluation of the primary endpoint ORR, 13 patients whose tumor response was not radiologically assessed due to early tumor-related death were classified as non-responders (8 in group A, 5 in group B). Five patients, two patients in group A and three patients in group B, did not undergo tumor response assessment due to other reasons. For these patients ORR was set to missing and imputed based on all other patients in their treatment group generating 10 imputed datasets and pooling the results using Rubins rule for analysis of the primary endpoint as described in [28]. These five patients were not included in regression analyses, resulting in a complete case set of 96 patients. With regard to the safety endpoints all patients were analyzed according to the treatment that they actually received, i.e., one patient allocated to group A who received nivolumab and died before radiotherapy initiation was analyzed in group B, resulting in 40 patients in group A and 61 patients in group B in the safety population (Fig. 1).

Patients in group A showed a significant enrichment for unfavorable parameters (*p* < 0.05), such as a worse ECOG status (ECOG 1; group A: 81%, group B: 58%), higher M status (M1c; group A: 71%, group B: 47%), and presence of extrathoracic metastases (group A: 90%, group B: 63%), representing mostly bone (group A: 65%, B: 37%) and skin (group A: 19%, group B: 3%) metastases (Table 1). Accordingly, bone lesions were the most frequently irradiated type of metastases in group A (55%). Furthermore, patients in group A showed numerically lower PD-L1 TPS values (*p* = 0.042–0.452 for various cut-off values, Table 1).

**Table 1:** Patients characteristics and distribution per treatment group

		Group A (nivolumab + RT) *n* = 41 (100%)	Group B (nivolumab only) *n* = 60 (100%)	*p *^*1*^
Sex	Male	24 (59%)	35 (58%)	0.984
Age (mean) ± SD		64.7 ± 7.2	67.7 ± 8.7	0.069 ^2^
smoking history		40 (98%)	52 (87%)	0.059
Therapy line	2	34 (83%)	54 (90%)	0.297
	3	7 (17%)	6 (10%)	
PD-L1 status	< 1%	20 (49%)	20 (33%)	0.104
	≥ 1%	18 (44%)	36 (60%)	
	< 5%	27 (66%)	28 (47%)	**0.042**
	≥ 5%	11 (27%)	28 (47%)	
	< 10%	27 (66%)	32 (53%)	0.171
	≥ 10%	11 (27%)	24 (40%)	
	< 50%	31 (82%)	42 (75%)	0.452
	≥ 50%	7 (18%)	14 (25%)	
	Missing*	3 (7%)	4 (7%)	
ECOG PS	0	8 (19%)	25 (42%)	**0.020**
	1	33 (81%)	35 (58%)	
CCI	≥ 2	9 (22%)	13 (22%)	0.973
SCS	> 9	7 (17%)	8 (13%)	0.604
M status ^3^	1a	4 (10%)	22 (37%)	**0.009**
	1b	8 (20%)	10 (17%)	
	1c	29 (71%)	28 (47%)	
Intrathoracic metastases		26 (64%)	45 (75%)	0.211
Extrathoracic metastases		37 (90%)	38 (63%)	**0.002**
	Skin	7 (19%)	1 (3%)	**0.022**
	Bone	24 (65%)	14 (37%)	**0.015**
	Lymph node	17 (46%)	17 (45%)	0.916
	Kidney	3 (8%)	3 (8%)	0.973
	Liver	9 (24%)	9 (24%)	0.948
	Adrenal gland	17 (46%)	20 (53%)	0.563
	Bone marrow	1 (3%)	1 (3%)	0.985
	Brain	9 (24%)	11 (29%)	0.651
	Spleen	1 (3%)	0 (0%)	0.308
	Other	4 (11%)	5 (13%)	0.754
Site of irradiation	Bone	22 (55%)	n/a	
	Skin	2 (5%)	n/a	
	Lymph node	7 (17.5%)	n/a	
	Bone & lymph node	1 (2.5%)	n/a	
	Other**	8 (20%)	n/a	

^1^ Chi^2^ test; ^2^ t-test; ^3^ according to UICC8; ECOG PS, Eastern Cooperative Oncology Group Performance Status; CCI, Charlson Comorbidity Index; RT, radiotherapy; SCS, Simplified Comorbidity Score. Significant p-values are shown in bold. * Local PD-L1 analyses were not conducted, and insufficient tumor material prevented central PD-L1 analyses. **Other includes kidney (1), soft tissue (2), pleura (1), thoracic wall (1), adrenal gland (2), liver (1)

### Efficacy

Among patients in group A, tumor assessment was performed for 39/41 patients, the two missing values of ORR were multiply imputed as described above. One patient achieved CR and two patients had a PR resulting in an ORR of 8.3% (95% CI [3.7%, 100%]) using multiple imputation. With the observed ORR below the expected 19%, the primary endpoint of this study was not met (*p* = 0.991 for one-sided binomial test) [5]. Eleven patients in group A had stable disease (SD) as best response (disease control rate [DCR] = 35.9%). Among 57 patients with response data in group B, one patient achieved CR, 12 patients had PR, and 20 patients had SD as best response, resulting in an observed ORR of 23.8% (95% CI [16.3%, 100%]) using multiple imputation, and a DCR of 57.9%. In the ITT population, ORR was inversely associated with the patients’ ECOG (OR 0.13, *p* = 0.004) and significantly correlated with PD-L1 expression (OR 12.8, *p* = 0.022), but not with RT administration (*p* = 0.854) (Table 2).

**Table 2:** Multivariate regression analyses adjusted for ECOG PS and PD-L1

	ORR^1^		PFS^2^		OS^2^	
	OR	*p*	HR	*p*	HR	*p*
Radiotherapy	0.861	0.854	1.367	0.169	1.219	0.452
ECOG PS	0.126	0.004	1.610	0.057	2.036	0.022
PD-L1 ≥ 1%	12.826	0.022	0.462	0.001	0.469	0.004

^1^: Logistic regression analysis performed in complete case set; ^2^: Cox regression analysis performed in ITT set. HR, Hazard Ratio; OR, Odds Ratio; ORR, objective response rate; PFS, progression-free survival; OS, median overall survival;

**Fig. 2:**
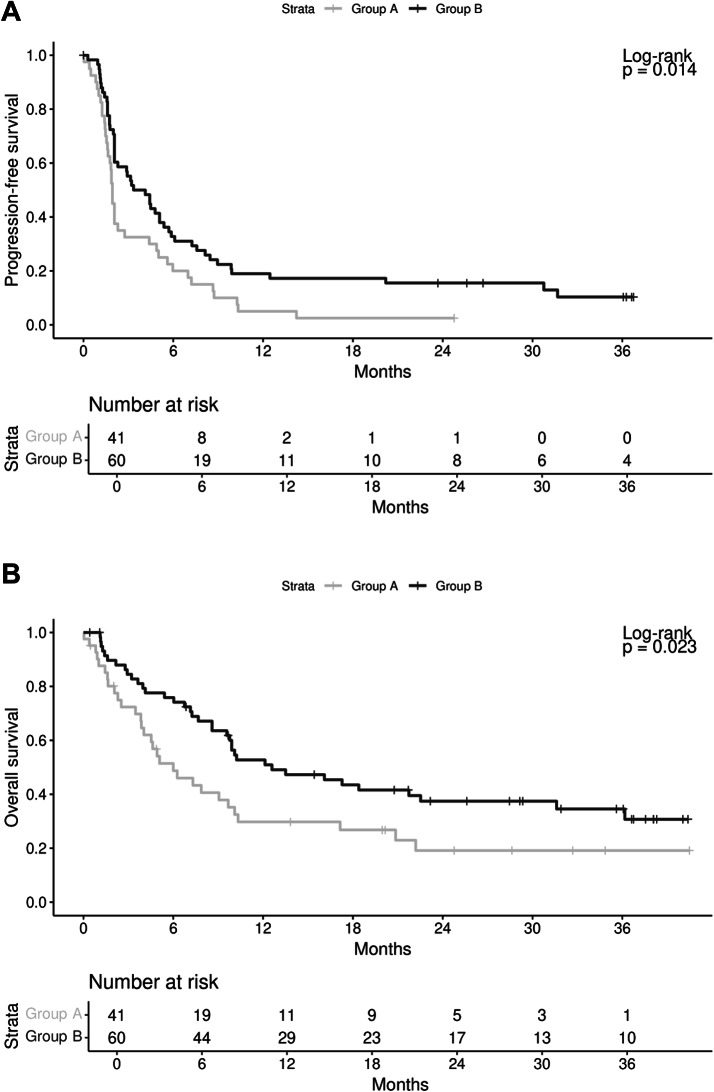
Kaplan–Meier estimates according to the two treatment groups of the FORCE trial. Group A received nivolumab + palliative radiotherapy, whereas group B was treated with only nivolumab. Analysis was performed starting from the first nivolumab infusion. (**A**) Progression-free survival, (**B**) Overall survival

PFS was significantly longer in group B with a 1-year PFS rate of 19.0% (95% CI [10.1%, 29.9%]) compared to 5.0% (95% CI [0.9%, 14.8%]) in group A (*p* = 0.014; HR 1.69 [95% CI (1.10, 2.58)]) (Fig. 2A). The median PFS in group A was 1.9 months (95% CI [1.6, 2.3]) and 3.7 months (95% CI [2.1, 5.4]) in group B. Similarly, OS was significantly longer in group B with a 1-year OS rate of 52.7% (95% CI [39.0%, 64.7%]) and a median OS of 12.6 months (95% CI [8.6, 31.6]) compared to a 1-year OS rate of 29.8% (95% CI [16.3%, 44.6%]) and a median OS of 6.0 months (95% CI [3.9, 10.1]) in group A (*p* = 0.023; HR 1.75 [95% CI (1.07, 2.84)]) (Fig. 2B).

**Fig. 3:**
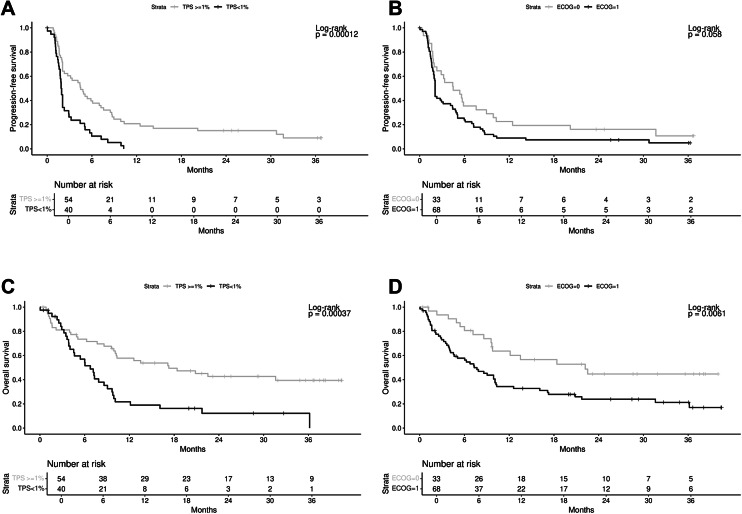
Kaplan–Meier estimates according to PD-L1 and ECOG in ITT population. (**A**) PFS in ITT population stratified according to PD-L1 TPS < 1% or ≥ 1%. (**B**) PFS in ITT stratified according to ECOG PS 0 or 1. (**C**) OS in ITT population stratified according to PD-L1 TPS < 1% or ≥ 1%. (**D**) OS in ITT population stratified according to ECOG PS 0 or 1

Survival analyses of ITT population revealed a significantly better outcome for patients with PD-L1 positive tumors and/or a better ECOG PS without significant effect from the administration of RT. Median PFS was 4.5 months (95% CI [2.1,7.0]) for patients with PD-L1 positive tumors versus 1.9 months (95% CI [1.6, 2.1], *p* = 0.00012; HR 0.4 [95% CI (0.3,0.7)]) with PD-L1 TPS < 1%, while median OS was 17.3 months (95% CI [9.9, n.r.]) versus 6.8 months (95% CI [4.0,9.0], *p* = 0.00037; HR 0.4 [95% CI (0.2,0.7)]), respectively (Fig. 3A and 3C and Supplementary Table 1). Besides, median PFS was 4.5 months (95% CI [1.9,7.6]) for patients with ECOG PS = 0 versus 2.1 months (95% CI [1.9,3.1]) for patient with ECOG PS = 1 (*p* = 0.058; HR 1.5 [95% CI (1.0, 2.4)], Fig. 3B), and median OS was 22 months (95% CI [9.7,n.r.] versus 7.3 months (95% CI [4.1,10.1], *p* = 0.0061; HR 2.2 [95% CI (1.2,3.8)], respectively (Fig. 3D).

On the other hand, multivariable analysis showed no significant association of RT administration with either PFS (HR = 1.367, *p* = 0.169) or OS (HR = 1.219, *p* = 0.452, Table 2).

Survival and objective response analyses of the two patient groups A (nivolumab with radiotherapy) and B (nivolumab only) according to PD-L1 status using different PD-L1 TPS cut-offs (1%, 5%, 10%, and 50%) revealed a significant association of PD-L1 expression with beneficial outcome in group B (Supplementary Table 1 and Supplementary Fig. 1). However, in the subgroup analysis of patients who have received combination therapy (group A) only a numerical improvement for PFS, OS, and ORR without statistical significance could be detected in PD-L1 high subgroups, probably due to the low patient number (Supplementary Table 1 and Supplementary Fig. 1).

No significant difference could be detected regarding PFS or OS according to the irradiated metastatic site for patients in group A [bone metastases (*n* = 22/21) versus other sites (lymph nodes, skin, and others; *n* = 18/19), Supplementary Table 2, Supplementary Fig. 2].

### Quality of life

In total, 670 post-baseline FACT-L questionnaires from 92 patients were collected. The physical well-being (PWB) score was significantly higher in patients who received immunotherapy only (group B) than in patients treated with both nivolumab and palliative radiotherapy, but the difference was small without clinical relevance (2.27 points, *p* = 0.018) (Supplementary Table 3, Supplementary Fig. 3A). For the remaining scores (SWB + EWB + FWB + LCS) no significant difference could be detected (difference between both groups were ≤ 1). Besides, the scores of the FACT-L total (PWB + SWB + EWB + FWB + LCS), FACT-G total (PWB + SWB + EWB + FWB), and the FACT-L trial outcome index (TOI) (PWB + FWB + LCS) were higher in group B compared to group A (3.86, 2.69, and 3.91 points, respectively), but these differences were not statistically significant. On the other hand, patients with higher scores at screening had significantly higher scores after screening for all sub- and total scales (0.51–0.63 points, *p* < 0.0001), which also indicated a key role of the baseline patient status for the subsequent clinical course, whether radiotherapy was given or not. For most subscales and total scales, no significant changes over time were detected, with the exception of SWB, which showed a slight reduction in QoL over time by mean 0.48 points per year (Supplementary Table 3). For the analyzed QoL scales, no significant changes over time were detected considering the entire group A when analysing the best change in FACT-L after receiving radiotherapy neither in the total scale nor in subscales (Supplementary Fig. 3B and not shown data). While for a proportion of patients in group A (8/27) a clinically relevant increase of ≥ 10 points on the FACT-L scale following RT could be measured, a comparable percentage of patients without an indication for RT (group B) (21/45) also demonstrated a clinically relevant increase of ≥ 10 points under treatment with immunotherapy as a monotherapy (Supplementary Fig. 3B).

### Safety

Almost all patients of the safety population experienced at least one AE according to CTCAE v4.03 during the study treatment (Table 3). Most common reported AEs of any grade included general disorders and administration site conditions (*n* = 144), respiratory, thoracic, and mediastinal disorders (*n* = 123), and gastrointestinal disorders (*n* = 115). The safety analysis did not reveal a significant difference between treatment groups, neither regarding the number of patients with grade 3–4 AEs nor regarding the number of documented grade 3–4 AEs per individual (*p* > 0.05). SAEs were defined as the occurrence of one or more of the following criteria: Death, life-threatening event, need for in-patient hospitalization, risk of disability or incapacity, or an important medical event jeopardizing the patient or requiring medical intervention to prevent one of the aforementioned outcomes. They occurred at least once in 58% and 56% of the patients in group A and group B, respectively. Two of the SAEs in group A were related to treatment (colitis due to nivolumab treatment; worsening general condition due to radiotherapy). In group B, eight of the SAEs were characterized as related to nivolumab administration (immune-related pneumonitis, 2 × increased gamma-glutamyl transferase, reduced general condition, 2 × palmar-plantar erythrodysesthesia syndrome, gastric hemorrhage, and ataxia).

**Table 3:** Documented (serious) adverse events according to CTCAE version 4.3 in both treatment groups

	Grade	Group A (nivolumab + RT) (*n* = 40)	Group B (nivolumab only) (*n* = 61)	*p*
Patients with at least one AE	All	40 (100%)	60 (98%)	0.416^1^
	3	23 (58%)	32 (53%)	0.619^1^
	4	1 (3%)	5 (8%)	0.236^1^
	5	3 (8%)	2 (3%)	0.339^1^
Patients with at least one SAE	All	23 (58%)	34 (56%)	0.861^1^
	3	15 (38%)	22 (36%)	0.884^1^
	4	1 (3%)	4 (7%)	0.358^1^
	5	3 (8%)	2 (3%)	0.339^1^

Number of treatment-related AEs grade 3–4	Group A (nivolumab + RT)	Group B (nivolumab only)	*p*
1	6 (86%)	5 (56%)	0.176^2^
2	1 (14%)	1 (11%)	
3	0	2 (22%)	
9	0	1 (11%)	

^1^: Chi^2^-test; ^2^: Mann–Whitney-U-test. AE, adverse event; RT, radiotherapy; SAE, serious adverse event

## Discussion

With a low ORR of 8% in the experimental arm, the FORCE phase II study did not meet the primary endpoint, which was tumor response in 19% of irradiated patients. Considering the clinical characteristics of study participants, this appeared to result mainly from the worse clinical condition of patients with an indication for palliative radiotherapy. Specifically, detrimental baseline parameters included higher ECOG status, higher tumor load, and a considerably higher number of patients with extrathoracic metastases, especially bone metastases, which have been shown to respond poorly to immunotherapy [29]. Furthermore, patients in group A showed a trend to lower PD-L1 TPS values (*p* = 0.042–0.452 for various cut-off values, Table 1), which was also associated with poorer response to ICI therapy in the current (Supplementary Table 1) and previous studies [30]. A clinically relevant immune-stimulatory synergy between nivolumab and ionizing radiation could not be demonstrated or may have been insufficient to counteract these negative prognostic factors. The low ORR was linked to significantly poorer baseline conditions of patients with a clinical indication for palliative radiotherapy, which could not be overcome by immunostimulatory effects. Indeed, ORR was significantly associated with the patients’ ECOG status (*p* = 0.004) and tumor PD-L1 expression (*p* = 0.022), but not with the administration of radiotherapy in multivariate analysis. PRO analysis of study patients using the FACT-L scale also suggested a key role of baseline status for subsequent clinical course with a significant correlation between initial and longitudinal values for all sub- and total scales regardless of the radiotherapy administration. The better clinical condition of patients in group B is also reflected in the significantly higher physical well-being score longitudinally (Supplementary Table 3), compared to group A, which may positively affect ICI efficacy in NSCLC [31], even though the magnitude of the difference was small. No significant changes over time were detected in any subscale or total score in neither group A or B, demonstrating the importance of careful selection of patients for palliative radiotherapy.

Of note, the frequency and severity of treatment-related adverse events did not differ between both groups either, and no treatment-related deaths were observed.

Retrospective studies analyzing the effect of adding radiotherapy to ICI have also had negative results, for example no improved survival using combined modality treatments in patients with advanced NSCLC treated with radiotherapy within three months of starting ICI or subsequently during ICI treatment [32], and no association of prior radiotherapy with nivolumab response in NSCLC [33]. Therefore, in order to avoid confounding by indication and assess the net effect of radiotherapy, a randomized comparison is of key importance. In a secondary analysis of the KEYNOTE-001 trial and one pooled analysis of two randomized trials (PEMBRO-RT phase II and MDACC phase I/II), for example, combination therapy (radiation + pembrolizumab) considerably improved outcomes of patients with advanced NSCLC [21, 34]. In detail, NSCLC patients who underwent combination therapy (radiotherapy + pembrolizumab) had longer OS (KEYNOTE-001: 10.7 months versus 5.3 months; PEMBRO-RT + MDACC: 19.2 months versus 8.7 months) and PFS (KEYNOTE-001: 4.4 months versus 2.1 months; PEMBRO-RT + MDACC: 9.0 months versus 4.4 months) than those who had not undergone radiotherapy [21, 34]. Both trials, however, included patients with less extrathoracic metastases than the FORCE trial, and the administered radiation doses were significantly higher. Furthermore, according to the previously mentioned study and the PACIFIC study, selecting an ideal time frame between irradiation and immunotherapy appears to be important (Bassanelli et al. study: ≤ 60 days before first nivolumab treatment; PACIFIC study: ≤ 2 weeks before first durvalumab treatment) for the therapeutic antitumor response brought on by the abscopal effect [35, 36]. In the current FORCE trial, nivolumab was initiated before radiotherapy. In a retrospective study, nivolumab treatment within 60 days before radiotherapy conclusion was found to improve median OS and PFS [35].

At the mechanistic level, radiotherapy promotes proinflammatory signals that trigger the innate immune system to activate tumor-specific T cells and causes the release of antigens following the death of cancer cells. It also upregulates immunogenic cell surface complex [37]. Mattes et al*.* [38] found that radiotherapy may be most beneficial when added to ICI monotherapy for patients with high tumoral PD-L1 expression. Evidence for significant immunologic activity was also evident in the FORCE trial, as PD-L1 expression correlated significantly with ORR and patient survival in the ITT. However, these effects could not overcome the profoundly worse clinical condition of patients with an indication for palliative radiotherapy. Along the same line, PD-L1 expression and clinical variables, like the ECOG status and metastatic burden, showed a stronger association with clinical benefit from nivolumab than the administration of additional radiotherapy in the FORCE trial, which should be considered in the prognostication of individual patients and the design of future radiotherapy/immunotherapy studies.

In conclusion, the combination of nivolumab and palliative radiotherapy was safe and feasible in the FORCE trial, but could not per se preserve or improve the clinical outcome for patients with clinical indication for palliative radiotherapy, who were characterized by a poorer baseline clinical condition compared to the non-irradiated group. Several trials, e.g. NCT03867175, NCT03774732, and NCT03110978, are currently ongoing to determine the optimal radiotherapy dose, timing, sequencing, and immunotherapy partner for radiotherapy in NSCLC.

## Supplementary Information

Below is the link to the electronic supplementary material.

Supplementary file1 (PDF 667 kb)

Supplementary file2 (DOCX 14 kb)

Supplementary file3 (DOCX 19 kb)

## Data Availability

The data underlying this article cannot be shared due to the privacy of individuals that participated in the study. However, deidentified individual participant data can be made available to others on acceptance of an official request to the corresponding author. The study protocol and other related documents can also be made available to others on request.

## Abbreviations

AE: Adverse event
CCI: Charlson comorbidity index
CI: Confidence interval
CONSORT: Consolidated standards of reporting trials
CR: Complete remission
CT: Computer tomography
DCR: Disease control rate
ECOG: Eastern Cooperative Oncology Group
EOS: End of study
EOT: End of protocol treatment
HR: Hazard ratio
ICI: Immune checkpoint inhibitors
LCS: Lung cancer subscale
MRI: Magnetic resonance imaging
NSCLC: non-small cell lung cancer
ORR: Objective response rate
OR: Odds ratio
OS: Overall survival
PFS: Progression-free survival
PR: Partial response
PRO: Patient-reported outcomes
RECIST: Response evaluation criteria in solid tumors
RT: Radiotherapy
SAE: Serious adverse event
SAP: Statistical analysis plan
SCS: Simplified comorbidity score
SD: Stable disease
TOI: Trial outcome index
TPS: Tumor proportion score

## References

[1] Siegel RL, Miller KD, Jemal A (2020) Cancer statistics, 2020. CA Cancer J Clin 70(1):7–30 31912902 10.3322/caac.21590

[2] Brahmer J, Reckamp KL, Baas P et al (2015) Nivolumab versus docetaxel in advanced squamous-cell non-small-cell lung cancer. N Engl J Med 373(2):123–135 26028407 10.1056/NEJMoa1504627 PMC4681400

[3] Reck M, Rodriguez-Abreu D, Robinson AG et al (2016) Pembrolizumab versus Chemotherapy for PD-L1-Positive Non-Small-Cell Lung Cancer. N Engl J Med 375(19):1823–1833 27718847 10.1056/NEJMoa1606774

[4] Remon J, Vilarino N, Reguart N (2018) Immune checkpoint inhibitors in non-small cell lung cancer (NSCLC): approaches on special subgroups and unresolved burning questions. Cancer Treat Rev 64:21–29 29454155 10.1016/j.ctrv.2018.02.002

[5] Borghaei H, Paz-Ares L, Horn L et al (2015) Nivolumab versus docetaxel in advanced nonsquamous non–small-cell lung cancer. N Engl J Med 373(17):1627–1639 26412456 10.1056/NEJMoa1507643 PMC5705936

[6] Hellmann MD, Rizvi NA, Goldman JW et al (2017) Nivolumab plus ipilimumab as first-line treatment for advanced non-small-cell lung cancer (CheckMate 012): results of an open-label, phase 1, multicohort study. Lancet Oncol 18(1):31–41 27932067 10.1016/S1470-2045(16)30624-6 PMC5476941

[7] Hellmann MD, Ciuleanu TE, Pluzanski A et al (2018) Nivolumab plus ipilimumab in lung cancer with a high tumor mutational burden. N Engl J Med 378(22):2093–2104 29658845 10.1056/NEJMoa1801946 PMC7193684

[8] Steuer CE, Ramalingam SS (2021) Advances in immunotherapy and implications for current practice in non-small-cell lung cancer. JCO Oncol Pract. 10.1200/OP.21.00305 34170753 10.1200/OP.21.00305

[9] Champiat S, Dercle L, Ammari S et al (2017) Hyperprogressive disease is a new pattern of progression in cancer patients treated by anti-PD-1/PD-L1. Clin Cancer Res 23(8):1920–1928 27827313 10.1158/1078-0432.CCR-16-1741

[10] De Ruysscher D, Reynders K, Van Limbergen E et al (2017) Radiotherapy in combination with immune checkpoint inhibitors. Curr Opin Oncol 29(2):105–111 28085679 10.1097/CCO.0000000000000352

[11] Takamori S, Toyokawa G, Takada K et al (2018) Combination therapy of radiotherapy and anti-PD-1/PD-L1 treatment in non-small-cell lung cancer: a mini-review. Clin Lung Cancer 19(1):12–16 28739315 10.1016/j.cllc.2017.06.015

[12] Adams DL, Adams DK, He J et al (2017) Sequential tracking of PD-L1 expression and RAD50 induction in circulating tumor and stromal cells of lung cancer patients undergoing radiotherapy. Clin Cancer Res 23(19):5948–5958 28679765 10.1158/1078-0432.CCR-17-0802

[13] Barker HE, Paget JT, Khan AA et al (2015) The tumour microenvironment after radiotherapy: mechanisms of resistance and recurrence. Nat Rev Cancer 15(7):409–425 26105538 10.1038/nrc3958 PMC4896389

[14] Formenti SC, Demaria S (2009) Systemic effects of local radiotherapy. Lancet Oncol 10(7):718–726 19573801 10.1016/S1470-2045(09)70082-8 PMC2782943

[15] Ngwa W, Irabor OC, Schoenfeld JD et al (2018) Using immunotherapy to boost the abscopal effect. Nat Rev Cancer 18(5):313–322 29449659 10.1038/nrc.2018.6 PMC5912991

[16] Ettinger DS, Wood DE, Akerley W et al (2015) Non-small cell lung cancer, version 6.2015. J Natl Compr Canc Netw 13(5):515–524 25964637 10.6004/jnccn.2015.0071

[17] Mole RH (1953) Whole body irradiation; radiobiology or medicine? Br J Radiol 26(305):234–241 13042090 10.1259/0007-1285-26-305-234

[18] Bhalla N, Brooker R, Brada M (2018) Combining immunotherapy and radiotherapy in lung cancer. J Thorac Dis 10(Suppl 13):S1447–S1460 29951296 10.21037/jtd.2018.05.107 PMC5994496

[19] Vatner RE, Cooper BT, Vanpouille-Box C et al (2014) Combinations of immunotherapy and radiation in cancer therapy. Front Oncol 4:325 25506582 10.3389/fonc.2014.00325 PMC4246656

[20] Fiorica F, Tebano U, Gabbani M et al (2021) Beyond abscopal effect: a meta-analysis of immune checkpoint inhibitors and radiotherapy in advanced non-small cell lung cancer. Cancers (Basel). 10.3390/cancers13102352 34068133 10.3390/cancers13102352 PMC8152785

[21] Shaverdian N, Lisberg AE, Bornazyan K et al (2017) Previous radiotherapy and the clinical activity and toxicity of pembrolizumab in the treatment of non-small-cell lung cancer: a secondary analysis of the KEYNOTE-001 phase 1 trial. Lancet Oncol 18(7):895–903 28551359 10.1016/S1470-2045(17)30380-7 PMC5538772

[22] Fiorica F, Belluomini L, Giuliani J et al (2021) Abscopal effect and resistance reversion in nivolumab-treated non-small-cell lung cancer undergoing palliative radiotherapy: a case report. Immunotherapy. 10.2217/imt-2021-0039 34180714 10.2217/imt-2021-0039

[23] Hotta T, Okuno T, Nakao M et al (2021) Reproducible abscopal effect in a patient with lung cancer who underwent whole-brain irradiation and atezolizumab administration. Thorac Cancer 12(6):985–988 33538129 10.1111/1759-7714.13875 PMC7952789

[24] Wang W, Huang C, Wu S et al (2020) Abscopal effect induced by modulated radiation therapy and pembrolizumab in a patient with pancreatic metastatic lung squamous cell carcinoma. Thorac Cancer 11(7):2014–2017 32391640 10.1111/1759-7714.13427 PMC7327675

[25] Theelen WS, de Jong MC, Baas P (2020) Synergizing systemic responses by combining immunotherapy with radiotherapy in metastatic non-small cell lung cancer: the potential of the abscopal effect. Lung Cancer 142:106–113 32126451 10.1016/j.lungcan.2020.02.015

[26] Bozorgmehr F, Hommertgen A, Krisam J et al (2019) Fostering efficacy of anti-PD-1-treatment: nivolumab plus radiotherapy in advanced non-small cell lung cancer - study protocol of the FORCE trial. BMC Cancer 19(1):1074 31703637 10.1186/s12885-019-6205-0 PMC6842256

[27] Gandhi L, Rodriguez-Abreu D, Gadgeel S et al (2018) Pembrolizumab plus Chemotherapy in Metastatic Non-Small-Cell Lung Cancer. N Engl J Med 378(22):2078–2092 29658856 10.1056/NEJMoa1801005

[28] Barnard J, Rubin DB (1999) Small-sample degrees of freedom with multiple imputation. Biometrika 86(4):948–955

[29] Landi L, D’Inca F, Gelibter A et al (2019) Bone metastases and immunotherapy in patients with advanced non-small-cell lung cancer. J Immunother Cancer 7(1):316 31752994 10.1186/s40425-019-0793-8 PMC6868703

[30] Horn L, Spigel DR, Vokes EE et al (2017) Nivolumab versus docetaxel in previously treated patients with advanced non-small-cell lung cancer: two-year outcomes from two randomized, open-label, phase III trials (CheckMate 017 and CheckMate 057). J Clin Oncol 35(35):3924–3933 29023213 10.1200/JCO.2017.74.3062 PMC6075826

[31] Zeng Y, Hu CH, Li YZ et al (2024) Association between pretreatment emotional distress and immune checkpoint inhibitor response in non-small-cell lung cancer. Nat Med 30(6):1680–1688 38740994 10.1038/s41591-024-02929-4 PMC11186781

[32] Samuel E, Lie G, Balasubramanian A et al (2021) Impact of radiotherapy on the efficacy and toxicity of anti-PD-1 inhibitors in metastatic NSCLC. Clin Lung Cancer 22(3):e425–e430 32778511 10.1016/j.cllc.2020.06.001

[33] Kataoka Y, Ebi N, Fujimoto D et al (2017) Prior radiotherapy does not predict nivolumab response in non-small-cell lung cancer: a retrospective cohort study. Ann Oncol 28(6):1402 28368440 10.1093/annonc/mdx114

[34] Theelen W, Chen D, Verma V et al (2021) Pembrolizumab with or without radiotherapy for metastatic non-small-cell lung cancer: a pooled analysis of two randomised trials. Lancet Respir Med 9(5):467–475 33096027 10.1016/S2213-2600(20)30391-X

[35] Bassanelli M, Ricciuti B, Giannarelli D et al (2022) Systemic effect of radiotherapy before or after nivolumab in lung cancer: an observational, retrospective, multicenter study. Tumori 108(3):250–257 33818208 10.1177/03008916211004733

[36] Antonia SJ, Villegas A, Daniel D et al (2017) Durvalumab after chemoradiotherapy in stage III non-small-cell lung cancer. N Engl J Med 377(20):1919–1929 28885881 10.1056/NEJMoa1709937

[37] Demaria S, Golden EB, Formenti SC (2015) Role of local radiation therapy in cancer immunotherapy. JAMA Oncol 1(9):1325–1332 26270858 10.1001/jamaoncol.2015.2756

[38] Mattes MD, Eubank TD, Almubarak M et al (2021) A prospective trial evaluating the safety and systemic response from the concurrent use of radiation therapy with checkpoint inhibitor immunotherapy in metastatic non-small cell lung cancer. Clin Lung Cancer 22(4):268–273 33608212 10.1016/j.cllc.2021.01.012 PMC8310528

