# Supplementary material for: Reconsidering palliative radiotherapy in addition to PD-1 blockade for non-small cell lung cancer: results from the FORCE phase II trial (AIO/YMO-TRK-0415)
Source: Clin Exp Metastasis. 2025 Jul 24;42(5):42. doi: 10.1007/s10585-025-10358-x (PMC12287132; doi:10.1007/s10585-025-10358-x)
Supplement: Supplementary file 1 — Supplementary file1 (PDF 667 kb) [file 10585_2025_10358_MOESM1_ESM.pdf]

Supplementary Figures

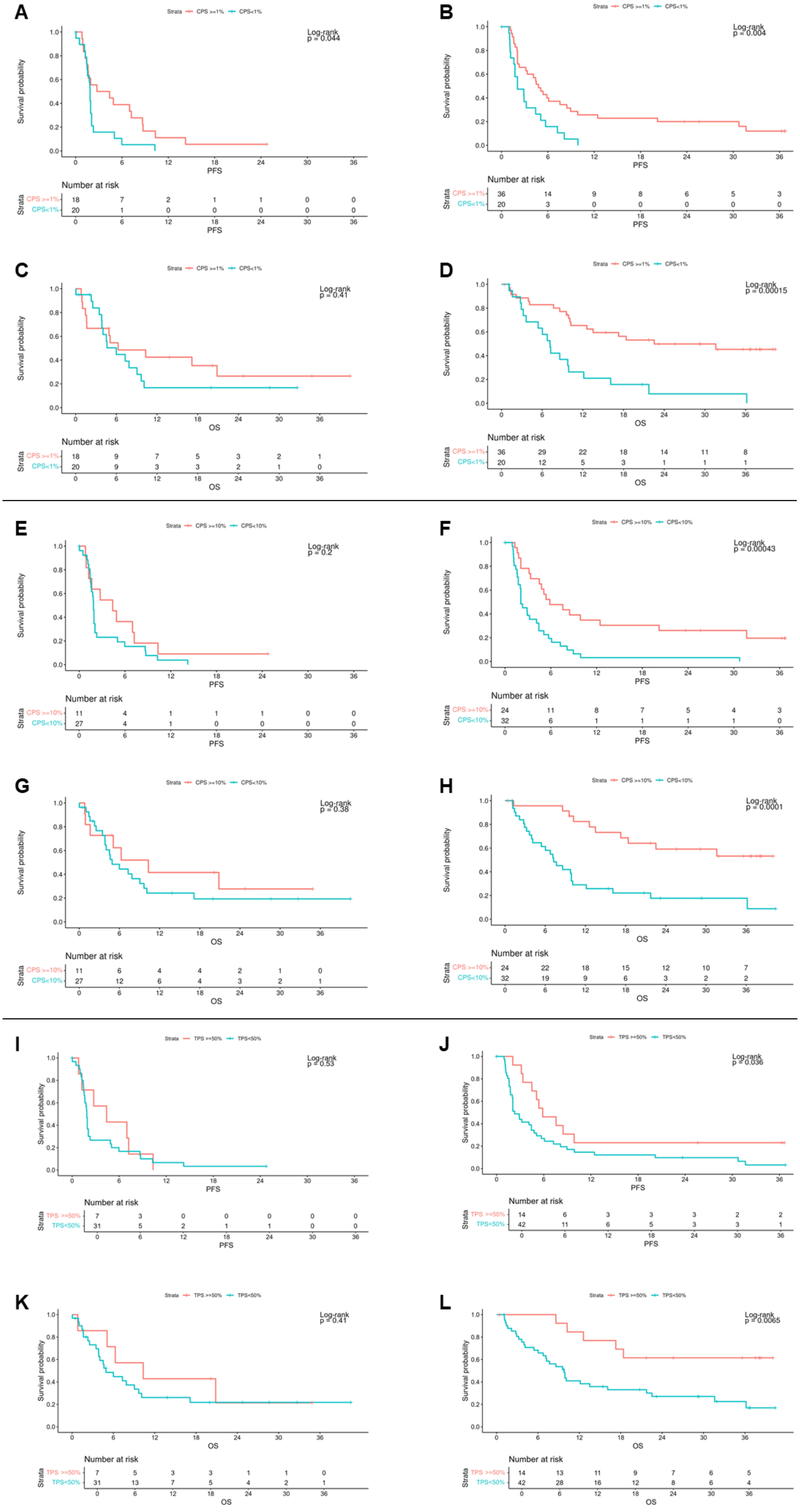

Supplementary Figure 1. Kaplan-Meier estimates according to PD-L1 subgroups in the FORCE treatment groups A (nivolumab + palliative radiotherapy) and B (nivolumab).

(A) PFS in patients treated with nivolumab + radiotherapy (group A) stratified according to PD-L1 TPS <1% or ≥1%.

(B) PFS in patients treated with nivolumab (group B) stratified according to PD-L1 TPS <1% or ≥1%.

(C) OS in patients treated with nivolumab + radiotherapy (group A) stratified according to PD-L1 TPS <1% or ≥1%.

(D) OS in patients treated with nivolumab (group B) stratified according to PD-L1 TPS <1% or ≥1%.

(E) PFS in patients treated with nivolumab + radiotherapy (group A) stratified according to PD-L1 TPS <10% or ≥10%.

(F) PFS in patients treated with nivolumab (group B) stratified according to PD-L1 TPS <10% or ≥10%.

(G) OS in patients treated with nivolumab + radiotherapy (group A) stratified according to PD-L1 TPS <10% or ≥10%.

(H) OS in patients treated with nivolumab (group B) stratified according to PD-L1 TPS <10% or ≥10%.

(I) PFS in patients treated with nivolumab + radiotherapy (group A) stratified according to PD-L1 TPS <50% or ≥50%.

(J) PFS in patients treated with nivolumab (group B) stratified according to PD-L1 TPS <50% or ≥50%.

(K) OS in patients treated with nivolumab + radiotherapy (group A) stratified according to PD-L1 TPS <50% or ≥50%.

(L) OS in patients treated with nivolumab (group B) stratified according to PD-L1 TPS <50% or ≥50%.

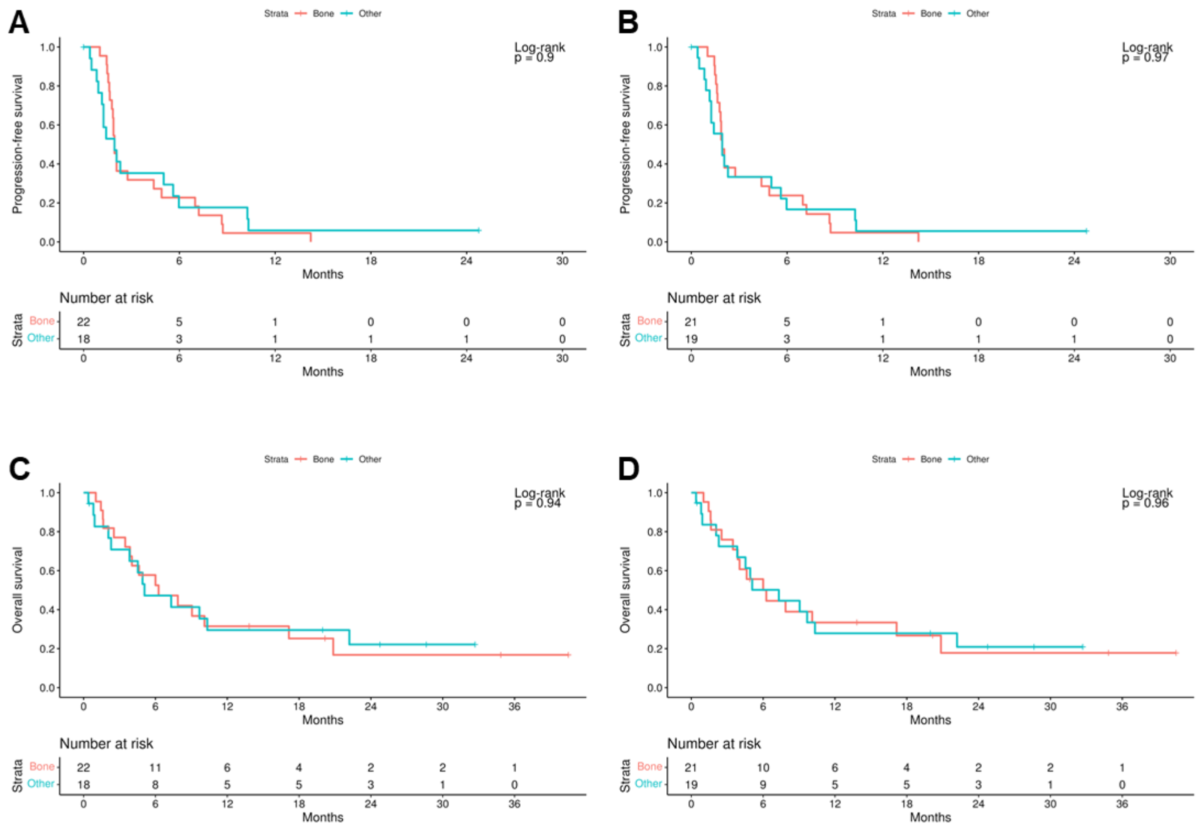

Supplementary Figure 2. Kaplan-Meier estimates according irradiated sites in treatment group A.

Two analyzed subgroups consisted of patients with irradiated metastatic lesions in either bones or other sites, including lymph nodes, skin and others. As one patient received irradiation at both bone and lymph node, separate analyses were performed assigning the patient either to the group irradiated at bone lesions or at other sites. One patient died before radiotherapy was initiated.

(A) PFS with one patient irradiated at two locations assigned to “bone” group,

(B) PFS with one patient irradiated at two locations assigned to “other” group,

(C) OS with one patient irradiated at two locations assigned to “bone” group,

(D) OS with one patient irradiated at two locations assigned to “other” groups.

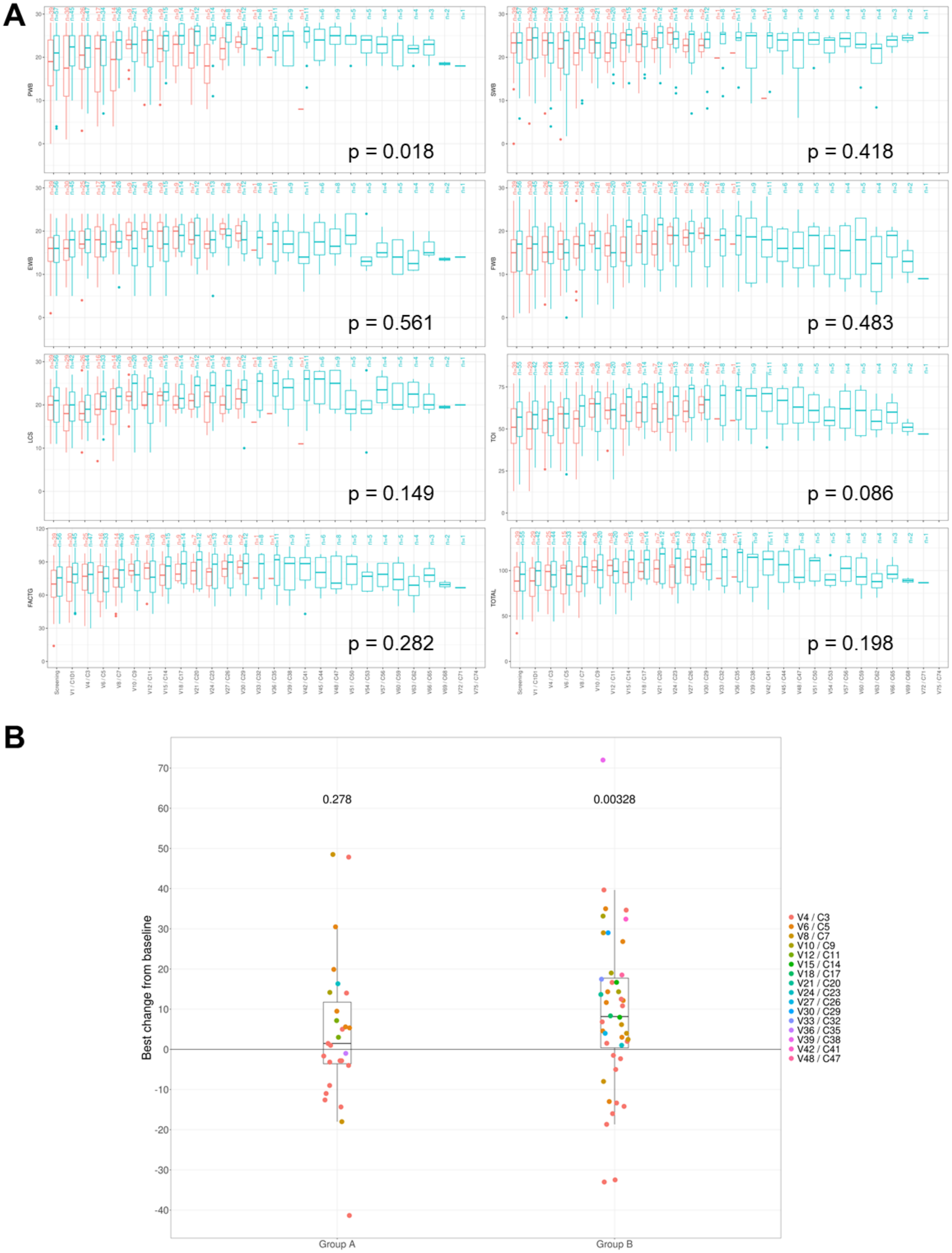
A
PWB
p = 0.018
SWB
p = 0.418
EWB
p = 0.561
FWB
p = 0.483
LCS
p = 0.149
TOI
p = 0.086
FACTG
p = 0.282
TOTAL
p = 0.198
B
Best change from baseline
0.278
0.00328
Group A
Group B
V4 / C3
V6 / C5
V8 / C7
V10 / C9
V12 / C11
V15 / C14
V18 / C17
V21 / C20
V24 / C23
V27 / C26
V30 / C29
V33 / C32
V36 / C35
V39 / C38
V42 / C41
V48 / C47

Supplementary Figure 3. Evolution of quality of life as assessed by the FACT-L questionnaire over time.

(A) Evolution of the scores of the five subscales of FACT-L (physical well-being [PWB], social/family well-being [SWB], emotional well-being [EWB], functional well-being [FWB] and lung cancer subscale [LCS]) as well as the FACT-L trial outcome index (TOI), the FACT-G total (FACT-G) and the FACT-L total score (FACT-L) over time. Red: group A, Blue: group B. P-values refer to average point difference group B over group A as assessed by the longitudinal mixed model (see Supplementary Table 3). C, cycle.

(B) Best change in FACT-L score from baseline for each patient, categorized by treatment group, with each dot representing one patient. Colors represent time points of occurrence of best change in FACT-L score from baseline. P-values were calculated using a one-sample t-test within each group to evaulate whether the mean change differed from zero. V, visit, C, cycle
