# Supplementary material for: Reconsidering palliative radiotherapy in addition to PD-1 blockade for non-small cell lung cancer: results from the FORCE phase II trial (AIO/YMO-TRK-0415)
Source: Clin Exp Metastasis. 2025 Jul 24;42(5):42. doi: 10.1007/s10585-025-10358-x (PMC12287132; doi:10.1007/s10585-025-10358-x)
Supplement: Supplementary file 2 — Supplementary file2 (DOCX 14 kb) [file 10585_2025_10358_MOESM2_ESM.docx]

***Supplementary Methods***

**Quality of life assessment**

Quality of life was assessed via the FACT-L questionnaire that consisted of five subscales: 1. physical well-being (PWB), 2. social/family well-being (SWB), 3. emotional well-being (EWB), 4. functional well-being (FWB), and 5. lung cancer subscale (LCS), and the patient’s functional ability was mapped on a five-point scale from 0-4.

**PD-L1 immunostaining**

PD-L1 immunohistochemistry was performed on an automated immunostainer (Ventana BenchMark Ultra, Ventana Medical Systems, Tucson, AZ, USA) using the biotin-free OptiView DAB IHC Detection Kit (Ventana Medical Systems, Oro Valley, AZ, USA). Three µm sections were cut from the formalin fixed and paraffin-embedded tissue blocks, deparaffinized, rehydrated and pre-treated with an antigen retrieval buffer (Tris/Borat/EDTA, pH 8.4). After blocking of endogenous peroxidase, the slides were incubated with monoclonal antibodies directed against PD-L1 (clone SP263, 1.61 µg/mL, Roche), followed by incubation with OptiView Universal Linker and OptiView HRP Multimer. PD-L1 staining of the tumor cells was analyzed and expressed as tumor proportion score (TPS), as defined before ^44^.

For 22 patients (10 in group A, 12 in group B), PD-L1 analysis was not possible due to insufficient tumor material. In these cases, local PD-L1 results were used for analysis. For 7 patients (3 in group A, 4 in group B), neither central nor local results were available.
