# Supplementary material for: Reconsidering palliative radiotherapy in addition to PD-1 blockade for non-small cell lung cancer: results from the FORCE phase II trial (AIO/YMO-TRK-0415)
Source: Clin Exp Metastasis. 2025 Jul 24;42(5):42. doi: 10.1007/s10585-025-10358-x (PMC12287132; doi:10.1007/s10585-025-10358-x)
Supplement: Supplementary file 3 — Supplementary file3 (DOCX 19 kb) [file 10585_2025_10358_MOESM3_ESM.docx]

***Supplementary Tables***

**Supplementary Table 1.** Progression-free survival, overall survival, and objective response rate (best overall response) in PD-L1 subgroups.

|  | **Med PFS, months (95% CI)** | | | | **Med OS, months (95% CI)** | | | | **ORR (n)** | | |
| --- | --- | --- | --- | --- | --- | --- | --- | --- | --- | --- | --- |
| **group** | **PD-L1 <1%** | **PD-L1 ≥1%** | ***p* ^1^** | **HR (95% CI)** | **PD-L1 <1%** | **PD-L1 ≥1%** | ***p* ^1^** | **HR (95% CI)** | **PD-L1 <1%** | **PD-L1 ≥1%** | ***p* ^2^** |
| **A** | 1.9 (1.4,2.1) | 3.6 (1.4,7.2) | 0.044 | 0.5 (0.2, 1.0) | 6.0 (3.8,9.1) | 6.3 (1.6, n.r.) | 0.414 | 0.7 (0.3, 1.6) | 5.3% (1) | 11.8% (2) | 0.481 |
| **B** | 2.1 (1.2,4.4) | 4.8 (2.3,7.6) | 0.004 | 0.4 (0.2, 0.8) | 7.2 (3.2,9.9) | 22.5 (10.2, n.r) | 0.0002 | 0.3 (0.1, 0.6) | 0% (0) | 32.4% (11) | 0.005 |
| **ITT*** | 1.9 (1.6,2.1) | 4.5 (2.1,7.0) | 0.0001 | 0.4 (0.3, 0.7) | 6.8 (4.0,9.0) | 17.3 (9.9, n.r) | 0.00037 | 0.4 (0.2, 0.7) | 2.6% (1) | 25.2% (13) | 0.003 |
|  |  |  |  |  |  |  |  |  |  |  |  |
|  | **PD-L1 <5%** | **PD-L1 ≥5%** | ***p* ^1^** |  | **PD-L1 <5%** | **PD-L1 ≥5%** | ***p* ^1^** |  | **PD-L1 <5%** | **PD-L1 ≥5%** | ***p* ^2^** |
| **A** | 1.9 (1.5,2.1) | 4.4 (0.9,7.2) | 0.203 | 0.6 (0.3, 1.3) | 4.9 (3.8,9.7) | 10.3 (0.9,n.r.) | 0.38 | 0.7 (0.3, 1.6) | 3.8% (1) | 20.0% (2) | 0.116 |
| **B** | 2.3 (1.7,4.4) | 5.1 (2.1,9.9) | 0.005 | 0.5 (0.3, 0.8) | 8.6 (5.4,12.1) | 31.6 (10.2,n.r.) | 0.0066 | 0.4 (0.2, 0.8) | 3.8% (1) | 37.0% (10) | 0.003 |
| **ITT*** | 2.0 (1.7,2.3) | 4.8 (2.8,7.2) | 0.0008 | 0.5 (0.3, 0.7) | 7.3 (4.6,9.8) | 20.9 (10.2,n.r.) | 0.0041 | 0.5 (0.3, 0.8) | 3.8% (2) | 32.4 % (12) | <0.001 |
|  |  |  |  |  |  |  |  |  |  |  |  |
|  | **PD-L1 <10%** | **PD-L1 ≥10%** | ***p* ^1^** |  | **PD-L1 <10%** | **PD-L1 ≥10%** | ***p* ^1^** |  | **PD-L1 <10%** | **PD-L1 ≥10%** | ***p* ^2^** |
| **A** | 1.9 (1.5,2.1) | 4.4 (0.9,7.2) | 0.203 | 0.6 (0.3, 1.3) | 4.9 (3.8,9.7) | 10.3 (0.9,n.r.) | 0.38 | 0.7 (0.3, 1.6) | 3.8% (1) | 20.0% (2) | 0.116 |
| **B** | 2.1 (1.7,4.1) | 5.9 (3.3,12.5) | 0.0004 | 0.4 (0.2, 0.6) | 7.3 (4.0,9.9) | n.r. (13.5,n.r.) | 0.0001 | 0.3 (0.1, 0.5) | 3.3% (1) | 43.5% (10) | <0.001 |
| **ITT*** | 2.0 (1.7,2.39 | 5.2 (3.1,8.5) | 0.0001 | 0.4 (0.3, 0.7) | 7.1 (4.1,9.7) | 31.6 (12.6,n.r.) | 0.0002 | 0.4 (0.2, 0.6) | 3.6% (2) | 36.4% (12) | <0.001 |

^1^: logrank-test; ^2^: Chi2-test; *: PD-L1 missing for 7 patients (3 in A, 4 in B), response missing for 5 patients (2 in A, 3 in B), resulting in an ITT-set of 94 patients for PFS and OS analysis, respectively, and 89 patients for ORR analysis.

HR, Hazard Ratio; med PFS, median progression-free survival; med OS, median overall survival; n.r. not reached; ORR, objective response rate;

**Supplementary Table 2.** Progression-free and overall survival in subgroups according to irradiation sites. Subgroups consisted of patients with irradiated metastatic lesions in either bones or other sites, including lymph nodes, skin and others. As one patient received irradiation at both bone and lymph node, separate analyses were performed assigning the patient either to the group irradiated at bone lesions or at other lesions. One patient died before radiotherapy was initiated.

|  | **bone metastasis**  **(*n*=22 / *n*=21)** | **other metastatic sites**  **(*n*=18 / 19)** |
| --- | --- | --- |
| **1-year PFS rate (95% CI) [%]** | 4.6 (0.3, 19.0) / 4.8 (0.3, 20.0) | 5.9 (0.4, 23.5) / 5.6 (0.4, 22.4) |
| **median PFS (95% CI) [months]** | 1.9 (1.6, 4.4) / 1.9 (1.6, 4.4) | 1.9 (0.9, 5.6) / 1.9 (1.2, 5.0) |
| ***p* ^1^** | 0.90 / 0.97 | |
| **HR (95% CI)** | 1.0 (0.5, 2.0) / 1.0 (0.5, 1.9) | |
| **1-year OS rate (95% CI) [%]** | 31.5 (13.1, 51.8) / 33.4 (14.0, 54.2) | 29.5 (10.8, 51.3) / 27.9 (10.2, 49.0) |
| **median OS (95% CI) [months]** | 6.3 (3.5, 17.2) / 6.3 (3.5, 17.2) | 5.1 (2.1, 22.2) / 7.3 (2.3, 10.3) |
| ***p* ^1^** | 0.94 / 0.96 | |
| **HR (95% CI)** | 1.0 (0.5, 2.1) / 1.0 (0.5, 2.1) | |

^1^: logrank-test

CI, confidence interval; HR, Hazard ratio; PFS, progression-free survival; OS, overall survival

**Supplementary Table 3.** Fixed effects from a longitudinal mixed model for FACT-L scores.

|  | **Average point difference group B over group A*** | ***p*** | **Average point difference per months*** | ***p*** | **number questionnaires** |
| --- | --- | --- | --- | --- | --- |
| Physical well-being (PWB) score | 2.27 ± 0.95 [0.39;4.14] | 0.018 | -0.006 ± 0.018  [-0.04;0.03] | 0.738 | 659 from 92 pts |
| Social well-being (SWB) score | -0.65 ± 0.81  [-2.24;0.93] | 0.418 | -0.040 ± 0.015  [-0.07;-0.01] | 0.008 | 657 from 92 pts |
| Emotional well-being (EWB) score | 0.35 ± 0.61  [-0.84;1.54] | 0.561 | 0.003 ± 0.015  [-0.03;0.03] | 0.828 | 647 from 91 pts |
| Functional well-being (FWB) score | 0.69 ± 0.98  [-1.24;2.62] | 0.483 | 0.040 ± 0.023  [-0.01;0.09] | 0.093 | 646 from 91 pts |
| Lung cancer subscale (LCS) score | 1.04 ± 0.71  [-0.36;2.43] | 0.149 | -0.021 ± 0.016  [-0.05;0.01] | 0.190 | 648 from 91 pts |
| FACT-L Trial outcome index (TOI) score (PWB+FWB+LCS) | 3.91 ± 2.27  [-0.56;8.37] | 0.086 | 0.002 ± 0.046  [-0.09;0.09] | 0.958 | 636 from 90 pts |
| FACT-G total score  (PWB+SWB+EWB+FWB) | 2.69 ± 2.50  [-2.22;7.59] | 0.282 | -0.003 ± 0.054  [-0.11;0.10] | 0.953 | 643 from 91 pts |
| FACT-L total score  (PWB+SWB+EWB+FWB+LCS) | 3.86 ± 2.99  [-2.02;9.73] | 0.198 | -0.031 ± 0.06  [-0.15;0.09] | 0.620 | 633 from 90 pts |

* estimated value ± standard error [lower; upper]; pts, patients
